# Incidence of tuberculosis in children on antiretroviral therapy: a retrospective cohort study

**DOI:** 10.1186/s13104-018-3846-z

**Published:** 2018-10-20

**Authors:** Aklilu Endalamaw, Eshetu Hailesilassie Engeda, Nega Tezera

**Affiliations:** Department of Pediatrics and Child Health Nursing, School of Nursing, College of Medicine and Health Sciences, University of Gondar, P.O.BOX 196, Gondar, Ethiopia

**Keywords:** Antiretroviral therapy, Children, HIV, Tuberculosis, Ethiopia

## Abstract

**Objectives:**

Be aware of the burden of tuberculosis among high-risk population is important. Three hundred fifty-two children were participated in this study. Survival analysis was conducted. We assessed the incidence of tuberculosis and its predictors in children on ART.

**Results:**

Tuberculosis incidence rate in children on ART was 2.63 per 100 person-years. Those children who were on baseline World Health Organization clinical stages 3 and 4 (AHR (adjusted hazard ratio) = 3.0; 95% CI 1.2–7.7), “fair” and “poor” ART adherence (AHR = 4.0; 95% CI 1.5–10.8), late initiation of ART (AHR = 4.0; 95% CI 1.5–10.6), and less than 6 months duration on ART (AHR = 5.5; 95% CI 1.5–20.6) were more likely to develop tuberculosis infection. The incidence rate of TB in children on ART was high. This study suggests a need to give attention to advanced AIDS stages and improve timely initiation of ART and level of adherence to ART.

## Introduction

Tuberculosis (TB) is one of the common causes of morbidity and mortality among human immunodeficiency virus positive patients (HIV) [1, 2], particularly in children [3, 4]. It is also listed among the top ten causes of death worldwide, in which one-third of all deaths of Acquired Immunodeficiency Syndrome (AIDS) are related to TB [5, 6].

According to 2016 World Health Organization (WHO) report [5], 10% of TB cases were children and people living with HIV. In Ethiopia, around 16,000 TB cases were found in HIV positive patients [7].

According to Sustainable Development Goal, there is a plan to reduce 80% of TB incidence by 2030. The “End TB” strategy has also set to stop TB epidemic through a process of reducing the number of TB deaths per year, TB incidence rate per year, and the catastrophic TB costs [8]. Initiation of antiretroviral therapy (ART) [9–14], cotrimoxazole prophylaxis therapy (CPT) [15], isoniazid prophylaxis therapy (IPT) [16–18], chemotherapy, tuberculosis vaccine, and early diagnosis [19] are help to prevent active TB infection in children.

Despite different interventions, TB incidence rate in children on ART is high in different settings at different time. Notably, 0.28 per 100 person-years reported in Latin America [16], 0.83 in China [20], 4.0 in South Africa [21], and 17.4 in East Africa [22].

Delayed motor development, underweight, WHO clinical stage 3 or 4 [23], CD4 (Cluster of Differentiation 4) cell count below threshold, anemia [24], and virological failure [25] are some of the contributing factors of TB incidence identified from previous studies.

In Ethiopia, information about the burden of new infection of TB in different settings is required [26]. This study can be used as an input to see the performance of activities implementing at district level based on 2030 end TB strategy progress evaluation. Besides, it helps to clinician to incorporate different predictors of active TB infection in HIV care clinic to the better prevention of the disease. Therefore, tracing TB among high-risk groups, like children on ART is important. This study was assessed the incidence of TB and its predictors in children on ART.

## Main text

### Methods

#### Study design, period, setting, and population

A retrospective cohort study from March 2005 to April 2017 was conducted at Debre-Markos Referral Hospital, northwest Ethiopia. Debre-Markos town is found in northwest Ethiopia 299 km from the capital Addis Ababa. According to the 2007 national census, this town has a total population of 62,497 [27]. It has one referral hospital, which serves more than 3.5 million people in its catchment area, where HIV care services have been provided since 2003. FMOH’s 2007 pediatric HIV/AIDS care guideline [28] was revised in 2015. Accordingly, regardless of the WHO clinical stage or CD4 cell count, all HIV positive children had started HAART since 2015.

All HIV positive children on ART were the study population. During the study period, 563 children were enrolled to ART Clinic. Of these, 352 children were included in this study.

Those children who had at least 1-month follow-up at the ART clinic were included. Children who were diagnosed to TB and those started anti-TB treatment at the beginning of the follow-up were excluded.

#### Operational definitions

##### Events

TB cases were identified based on TB diagnosis guideline of the Ethiopian Ministry of Health [28], using sputum or gastric aspirate microscopy, chest X-ray examination, and/or histopathology.

##### Censored

Lost, drop out, transfer out, died of other causes or completed study period before developing TB.

##### TB-free probability time

Considered between ART starting and TB diagnosis date.

##### Level of ART adherence

Good (≥ 95% or < 2 doses missed per month or < 3 dose missed per 2 months), fair (85–94% or 3–5 doses missed per 30 doses or 3–9 doses of 60 doses), and poor (less than 85% or > 6 doses of 30 doses or > 9 dose of 60 doses) [29].

##### Underweight

According to WHO curve, weight for age Z score < − 2 standard deviation.

##### CD4 cell count below threshold

CD4 cell count < 1500/mm^3^ (< 25%) for < 12 months, CD4 cell count < 750/mm^3^ (< 20%) for age 12–35 months, CD4 cell count < 350/mm^3^ (< 15%) for age 36–59 months, and CD4 cell count < 200/mm^3^ (< 15%) for age ≥ 60 months [30].

#### Data collection tools, procedures, and quality control

Data were collected using the data abstraction tool prepared from WHO ART follow-up chart. The medical record numbers of HIV-positive children who started ART were taken from computer records. Then, charts were drawn from the patients’ medical chart room. Three clinical nurses who took ART training were collected the data. The overall activities and the aim of the study were informed to data collectors. Questions raised by data collectors were explained to create better understanding. The principal investigator and one supervisor followed data collection process. Consistency of data was checked by random selection of five percent of the extracted data tool to cross-check with the medical charts of children. Each chart was coded to avoid the duplication of data.

#### Data processing and analysis

Data was entered into EPI data 3.1 and exported to STATA version 12 statistical package software for analysis. Summary statistics were applied to describe the study population in relation to studied variables. TB incidence rate was determined per 100 person-years.

Kaplan–Meier survival function was used to estimate TB-free probability. Those variables had reported p-value < 0.2 in the log-rank test were entered into the multivariable Cox-proportional model. The Cox-proportional hazards model assumption was checked for variables in the final model by the Schoenfeld residual test. None of variables broke the proportional assumption model. An adjusted hazard ratio with a 95% confidence interval (CI) was estimated. Those variable had p-value below 0.05 in the multivariable analysis was considered statistically significant predictors of TB infection.

#### Ethical considerations

The School of Nursing on behalf of the University of Gondar Institutional Ethical Review Board issued the ethical clearance. A written permission was obtained from the administrative office of Debre-Markos Referral Hospital and wrote a letter of permission to ART focal person. The names and/or identification numbers of patients were not recorded on the data extraction tool. All data were kept strictly confidential and used only for the study purposes.

### Results

#### Demographic, clinical, laboratory, and medication-related characteristics

Three hundred fifty-two HIV positive children on ART were included in this study. The mean age of the study participants was 6.73 (standard deviation = 3.84) years. Majority (43.7%) of them were found in the age group of 5–10 years. Slightly more than half (50.9%) of the children were male and nearly 90% from urban area. For more than two-thirds (70.5%) of children’s parents were both alive. Majority (78.1%) of children were on WHO clinical stage 1 and 2. 80.4% had above threshold CD4 cell count. About 14.2% had < 10 mg/dl hemoglobin level, 22.3% were underweight, 94.0%, and 31.2% of children were taking CPT and IPT, respectively. Majority (82.4%) of children were initiated ART lately. Children who had good adherence level to ART and on ART for greater than 6 months were 85.5% and 84.1%, respectively (Table 1).

**Table 1:** Baseline demographic, clinical, laboratory and medication-related characteristics of children on ART at Debre-Markos Referral Hospital, Northwest Ethiopia from March 2005 to April 2017 (n = 352)

Characteristics	Categories	Frequency	Percent (%)
Age	0–5 year	140	39.8
	5–10 year	154	43.7
	10–15 year	58	16.5
Sex	Male	179	50.9
	Female	173	49.1
Residence	Urban	313	88.9
	Rural	39	11.1
Parent status	Both parents alive	248	70.5
	Paternal and maternal orphan	80	22.7
	Double orphan	24	6.8
WHO clinical stage	Stage 1 and 2	275	78.1
	Stage 3 and 4	77	21.9
CD4 count	Below threshold	69	19.6
	Above threshold	283	80.4
Hemoglobin level	< 10 mg/dl	50	14.2
	≥ 10 mg/dl	302	85.8
Nutritional status	Underweight	82	22.3
	Normal	270	76.7
Ever taking cotrimoxazole preventive therapy	Yes	331	94.0
	No	21	6.0
Ever taking isoniazid prophylaxis therapy	Yes	110	31.2
	No	242	68.8
Months on care before ART	≤ 3 month	290	82.4
	> 3 months	62	17.6
ART adherence	Good	301	85.5
	Ever had fair and poor	51	14.5
Months on care after ART	≤ 6 month	56	15.9
	> 6 month	296	84.1

*ART* antiretroviral therapy, *WHO* World Health Organization

#### Tuberculosis incidence rate during follow-up

A follow-up was conducted from 1 to 143 months on 352 participants yielded 1294.7 years-time at risk. New TB cases were observed in 34 (9.7%) of study participants making the overall TB incidence rate 2.63 (95% CI 1.9–3.7) per 100 person-years.

#### Kaplan–Meier TB-free probability

**Fig. 1:**
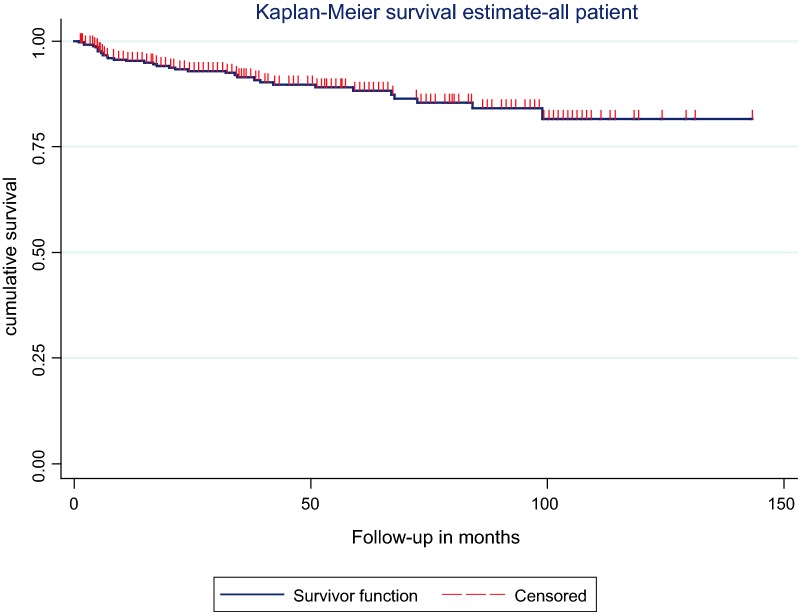
Kaplan-Meier estimate of TB-free probability in children on ART at Debre-Markos Referral Hospital, Northwest Ethiopia from March 2005 to April 2017

The median TB-free probability time was 135 months. TB-free probability by the end of the follow-up was 0.82 (95% CI 0.76–0.87) (Fig. 1).

#### Predictors of TB incidence in HIV positive children on ART

In the multivariable Cox-regression, WHO clinical stage, initiation time of ART, level of adherence to ART, and duration on ART were significant predictors of TB incidence.

Children on WHO clinical stages 3 and 4 were 3 times (AHR = 3.0, 95% CI 1.2–7.7) more risk of getting TB infection than those on WHO clinical stages 1 and 2. “Fair” and “poor” adherence level to ART was 4 times (AHR = 4.0, 95% CI 1.5–10.8) more risk of developing TB compared to “good” ART adherence. Late initiation of ART was 4 times (AHR = 4.0, 95% CI 1.5–10.6) higher risk of developing TB than starting otherwise. Children who took ART for less than or equal to 6 months were 5.5 times (AHR = 5.5, 95% CI 1.5–20.6) higher risk of TB infection than their counterparts (Table 2).

**Table 2:** Cox-proportional hazard analysis of predictors of TB incidence in children on ART at Debre-Markos Referral Hospital, Northwest Ethiopia from March 2005 to April 2017

Variables	Bivariable		Multivariable	
	p value	HR (95% CI)	p value	HR (95% CI)
Age				
0–5 year	0.860	1.1 (0.4, 3.1)		
5–10 year	0.861	1.1 (0.4, 3.0)		
10–15 year		1		
Sex				
Male		1		
Female	0.428	1.4 (0.7, 2.6)		
Residence				
Urban		1		1
Rural	0.051	2.3 (1.0, 5.3)	0.333	1.7 (0.6, 5.0)
Parent status				
Both parents alive		1		1
Paternal/maternal orphan	0.008	2.7 (1.3, 5.7)	0.875	0.9 (0.4,2.3)
Double orphan	0.010	3.8 (1.4, 10.3)	0.434	0.6 (0.2,2.1)
WHO clinical stage				
Stage 1 and 2		1		1
Stage 3 and 4	< 0.001	11.6 (5.5, 24.3)	0.020	3.0 (1.2, 7.7)^*^
CD4 count				
Below threshold	0.001	3.4 (1.7, 7.0)	0.157	1.8 (0.8,4.2)
Above threshold		1		1
Hemoglobin				
< 10 mg/dl	0.001	3.5 (1.7, 7.3)	0.345	1.6 (0.6,3.9)
≥ 10 mg/dl		1		1
Nutritional status				
Underweight	0.003	2.8 (1.4, 5.5)	0.757	1.1 (0.5,2.5)
Normal		1		1
Ever taking CPT				
Yes		1		1
No	< 0.001	7.2 (3.1,16.8)	0.507	1.4 (0.5,3.9)
Ever taking IPT				
Yes		1		1
No	0.025	3.0 (1.1,7.7)	0.870	1.1 (0.4,3.1)
Months on care before ART				
≤ 3 month		1		1
> 3 month	< 0.001	12.6 (6.2,25.7)	0.006	4.0 (1.5,10.6)^*^
ART adherence				
Good		1		1
Ever had fair and poor	< 0.001	17.4 (8.4,35.9)	0.005	4.0 (1.5,10.8)^*^
Months on care after ART				
≤ 6 month	< 0.001	13.1 (5.2,33.1)	0.011	5.5 (1.5,20.6)^*^
> 6 month		1		1

*HR* hazard ratio, *CI* confidence interval *independently significant at α 0.05

### Discussion

This study aimed to assess TB incidence rate and its predictors in children on ART. Accordingly, the overall TB incidence rate was 2.63 (95% CI 1.9–3.7) per 100 person-years. This finding was comparable to that of a study conducted in south Ethiopia [31]. This might be due to similar socio-economic characteristics, HIV care settings, and HIV care guideline.

On the other hand, TB incidence rate of this study was higher than that of a study conducted in Latin America [16], UK and Ireland [32], and China [20]. This variation might be due to the fact that the incidence rate of TB was low in the general population of high-income countries. Besides, the presence of advanced technologies, implementation of early TB diagnosis and prevention strategies in developed countries play a significant role to reduce TB incidence [33]. Other possible reasons, like poverty, crowding, many family members, and poor housing conditions contribute to a higher TB incidence rate in Ethiopia.

In the current study, WHO clinical stage, level of ART adherence, late initiation of ART, and duration on ART were associated factors of TB incidence. This study showed that the incidence of TB was higher among WHO clinical stages 3 and 4 compared to 1 and 2. This finding was in agreement with those of studies done in Tanzania [24], China [20], UK, and Ireland [32]. The declining of immunity in advanced WHO clinical stages accelerates the progression of latent TB infection to active TB infection so that children presenting with advanced WHO clinical stages need to have close monitoring.

It is encouraging that early initiation of ART [34] to be one of the relevant strategies for the prevention of OIs, like TB. Initiation of ART early after confirmation of HIV infection even at higher CD4 cell count helps to reduce the occurrence of TB [35]. In our study, those children initiated ART lately were more likely to develop TB infection. Other studies in Tanzania [24] and Kenya [36] also revealed that the risk of TB infection was higher among children who started ART lately.

ART is continuing to prevent TB among HIV positive patients [37]. Having “fair” and “poor” adherence level to ART creates a suitable environment for viral replication and can facilitate the emergence of ART resistance, which could leads to an increased viral load, and a declining immunological status and opportunistic infections become prevalent as a result. This study established “fair” and “poor” ART adherence level was found to be a risk factor of TB incidence.

In the current study, children who took ART for less than or equal to 6 months were at higher risk of developing TB compared to those who took ART for more than 6 months. Similar studies conducted in Kenya [9, 38], Uganda and Zimbabwe [39], Tanzania [24], Cote D’Ivoire [40], and South Africa [21] were concordant with ours. A possible explanation was children might have had a non-symptomatic TB at the time of ART initiation. Moreover, patients may come up with other severe co-OIs which might divert the attention of health professionals to control other OIs. Furthermore, patients who started ART might experience changes in clinical manifestations due to the unmasking of clinical signs and symptoms [41].

TB continues to be the public health agenda in Ethiopia. Therefore, prevention of TB through strengthening of TB diagnostic approaches and implementation of strong preventive strategies to children on ART has been suggested. Notably, newly emerging prophylaxis regimen in the prevention of TB [42] needs to be considered. More emphasis is required for the first 6 months after ART initiation. Parents and/or caregivers should be incorporated into HIV care settings to retain children on ART regimens. In Ethiopia, due to many reasons, children might not start ART soon after confirmation of HIV status. Initiation of early childhood diagnosis and screening in the community need more emphasis to identify them before advanced disease stages.

## Limitation of the study

This study was retrospective follow-up and depends on medical records, which are not designed for research; the small numbers of children in some exposure categories; the reliance on clinical diagnosis for most children and the potential for misclassification that arises from this. TB incidence might be underestimated due to excluded of charts with incomplete data. Variables, like income, housing condition, caregiver, family size, and viral load were not investigated. This study took nearly a 12-year period, during which there were likely several changes to clinical practice. The time-covariate variable was not handled because the data were collected at baseline.

## Abbreviations

AIDS: Acquired Immunodeficiency Syndrome
AHR: adjusted hazard ratio
ART: highly active antiretroviral therapy
CD4: Cluster of Differentiation 4
CI: confidence interval
CPT: cotrimoxazole prophylactic therapy
DRC: Democratic Republic of Congo
HIV: human immunodeficiency virus
IPT: isoniazid prophylactic therapy
OI: opportunistic infection
TB: tuberculosis
UK: United Kingdom
WHO: World Health Organization
